# CD163 macrophage and erythrocyte contents in aspirated deep vein thrombus are associated with the time after onset: a pilot study

**DOI:** 10.1186/s12959-016-0122-0

**Published:** 2016-11-22

**Authors:** Eiji Furukoji, Toshihiro Gi, Atsushi Yamashita, Sayaka Moriguchi-Goto, Mio Kojima, Chihiro Sugita, Tatefumi Sakae, Yuichiro Sato, Toshinori Hirai, Yujiro Asada

**Affiliations:** 1 Department of Radiology, Faculty of Medicine, University of Miyazaki, 5200 Kihara, Kiyotake, Miyazaki 889-1692 Japan; 2 Department of Pathology, Faculty of Medicine, University of Miyazaki, 5200 Kihara, Kiyotake, Miyazaki 889-1692 Japan; 3 Department of Diagnostic Pathology, Miyazaki University Hospital, University of Miyazaki, 5200 Kihara, Kiyotake, Miyazaki 889-1692 Japan; 4 Department of Biochemistry and Microbiology, Faculty of Pharmaceutical Sciences, Kyusyu University of Health and Welfare, 1714-1 Yoshinomachi, Nobeoka, 882-0072 Japan

**Keywords:** CD163, Deep vein thrombus, Erythrocyte, Macrophage

## Abstract

**Background:**

Thrombolytic therapy is effective in selected patients with deep vein thrombosis (DVT). Therefore, identification of a marker that reflects the age of thrombus is of particular concern. This pilot study aimed to identify a marker that reflects the time after onset in human aspirated DVT.

**Methods:**

We histologically and immunohistochemically analyzed 16 aspirated thrombi. The times from onset to aspiration ranged from 5 to 60 days (median of 13 days). Paraffin sections were stained with hematoxylin and eosin and antibodies for fibrin, glycophorin A, integrin α2bβ3, macrophage markers (CD68, CD163, and CD206), CD34, and smooth muscle actin (SMA).

**Results:**

All thrombi were immunopositive for glycophorin A, fibrin, integrin α2bβ3, CD68, CD163, and CD206, and contained granulocytes. Almost all of the thrombi had small foci of CD34- or SMA-immunopositive areas. CD68- and CD163-immunopositive cell numbers were positively correlated with the time after onset, while the glycophorin A-immunopositive area was negatively correlated with the time after onset. In double immunohistochemistry, CD163-positive cells existed predominantly among the CD68-immunopositive macrophage population. CD163-positive macrophages were closely localized with glycophorin A, CD34, or SMA-positive cell-rich areas.

**Conclusions:**

These findings indicate that CD163 macrophage and erythrocyte contents could be markers for evaluation of the age of thrombus in DVT. Additionally, CD163 macrophages might play a role in organization of the process of venous thrombus.

**Electronic supplementary material:**

The online version of this article (doi:10.1186/s12959-016-0122-0) contains supplementary material, which is available to authorized users.

## Background

Venous thromboembolisms are a global health problem. The annual incidence of venous thromboembolism ranges from 0.75 to 2.69 per 1000 individuals in Western Europe, North America, Australia, and Argentina. This incidence has recently increased to between 2 and 7 per 1000 among those aged 70 years or older [1]. In a series of 140 cases of fatal pulmonary embolisms, the emboli originated in the iliac veins (4.5%), femoral veins (20.7%), and deep crural veins (74.8%) [2]. The pathological findings of venous thrombus vary with the time after onset. Pathological findings include platelet aggregation and fibrin formation with erythrocytes and neutrophils, lysis of cellular components, endothelial reactions and fibroblast proliferation, and replacement of thrombi with fibrous tissue and formation of recanalized vessels. Findings of deep vein thrombosis (DVT) have been obtained from pathological and forensic examinations, and are affected by degenerative changes at postmortem, therapeutic effects, and circulatory disturbance in the agonal stage.

Thrombolytic therapy can be more effective in selected patients with DVT compared with anticoagulant treatment alone [3, 4]. Addition of catheter-directed thrombolysis in conventional anticoagulant treatment for acute DVT reduces the risk of post-thrombotic syndrome by 14% at a 2-year follow-up and 28% at a 5-year follow up [5, 6]. Thrombolytic therapy results in a substantial increase in the risk of bleeding. Therefore, identification of a marker that reflects the age of thrombus is of particular concern. Alternatively activated macrophages are related to resolution of inflammation, tissue repair, and angiogenesis [7]. Therefore, we hypothesize that alternatively activated macrophages reflect the age of thrombus, as well as endothelial and fibroblastic proliferation.

Aspiration thrombectomy has been used to restore vascular patency alone and in conjunction with thrombolytic agents [8]. For treatment of acute DVT, aspiration thrombectomy with or without stenting is superior to anticoagulant therapy alone in terms of ensuring venous patency and improving clinical symptoms [9]. Technological advances have allowed evaluation the pathological changes in culprit thrombi in patients with DVT.

This pilot study aimed to determine the cellular and molecular components, and to identify a marker that reflects the time after onset in human aspirated DVT.

## Methods

### Aspirated thrombi from patients with DVT

**Fig. 1:**
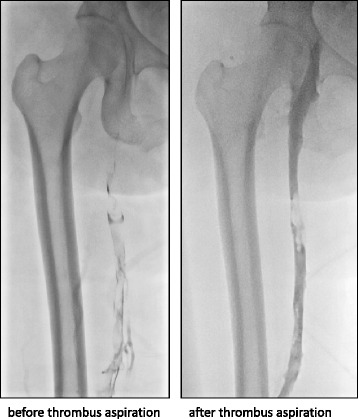
Representative images of X-ray venography before and after thrombus aspiration. X-ray venography shows an extensive filling defect before thrombus aspiration along the femoral vein, and a considerable reduction in the defect after thrombus aspiration



The ethics committee of the Miyazaki University approved the study protocol (approval no. 427). We obtained informed consent from the patients with DVT. Sixteen thrombi were obtained from 16 patients (8 men and 8 women; age range, 35–78 years) with DVT who were diagnosed based on clinical symptoms, laboratory data, and clinical imaging findings. The thrombi were mainly aspirated from the proximal portion of the thrombi with a guiding catheter (Guider Softip Guiding Catheter; Boston Scientific Japan, Tokyo, Japan). This catheter was placed from the popliteal vein to the iliac vein (10 cases), the leg vein to the inferior vena cava (5), and the subclavian vein to the superior vena cava (1). X-ray venography showed an extensive filling defect in the femoral vein before thrombus aspiration and reduction of the filling defect after thrombus aspiration (Fig. 1). An additional movie file shows this in more detail (see Additional file 1). We estimated the time of onset based on clinical symptoms and a medical interview. The times from onset to aspiration varied from 5 to 60 days, with a median of 13 days. The underlying diseases of the patients included the following: trauma and long-term immobilization (*n =* 5), idiopathic (*n =* 3), malignant tumor postoperatively (*n =* 2), chronic inflammatory disease (*n =* 2), Budd–Chiari syndrome (*n =* 1), chronic renal failure (*n =* 1), peripheral artery disease (*n =* 1), and gynecologic surgery (*n =* 1). Thirteen of 16 patients were intravenously administered heparin for 1 to 30 days, and six of 16 patients were intravenously administered thrombolytic agents for 1 to 12 days before thrombus aspiration.

**Additional file 1**: Additional movie file. Representative movie of X-ray venography during thrombus aspiration. An aspiration catheter was introduced in a thrombotic femoral vein and placed at the proximal portion of the thrombus. The venous thrombus was aspirated under negative pressure in a retrograde manner


All aspirated thrombi were immediately fixed in 4% paraformaldehyde and embedded in paraffin for histological evaluation. Sections (4-μm thick) were stained with hematoxylin and eosin and morphologically assessed. Consecutive sections were immunohistochemically analyzed.

### Immunohistochemistry

Consecutive 4-μm slices were immunohistochemically stained using antibodies against α-smooth muscle actin (SMA) (mouse monoclonal, clone 1A4; Dako, Glostrup, Denmark), CD34 (mouse monoclonal, QBEnd 10; Dako), CD68 (mouse monoclonal, clone PGM-1; Dako), CD163, a macrophage scavenger receptor (mouse monoclonal, clone 10D6; Leica Microsystems, Newcastle upon Tyne, UK), CD206, mannose receptor C type 1 (mouse monoclonal, clone 5C11; LifeSpan Biosciences, Inc., Seattle, WA, USA), fibrin (mouse monoclonal, clone T2G1; Accurate Chemical & Scientific Corp., Westbury, NY, USA), glycophorin A (mouse monoclonal, clone JC159; Dako), and platelet integrin α2bβ3 (sheep polyclonal; Affinity Biologicals Inc., Hamilton, CA, USA). The sections were stained with Envision (DAKO) or donkey anti-sheep IgG secondary antibody (Jackson ImmunoResearch, Baltimore, MA, USA). Horseradish peroxidase activity was visualized using 3, 3′-diaminobenzidine tetrahydrochloride. The immunostaining controls included non-immune mouse or sheep IgG instead of primary antibodies.

We performed double immunohistochemical staining for CD163 (rabbit polyclonal; DB Biotech, Kosice, Slovakia) and CD68 (Dako), CD163 (DB Biotech) and CD34 (Dako), CD163 (DB Biotech) and glycophorin A (Dako), and CD163 (DB Biotech) and SMA (Dako) using the MACH2 Double Stain kit (Biocare Medical, Concord, CA, USA).

### Quantitative methods

**Fig. 2:**
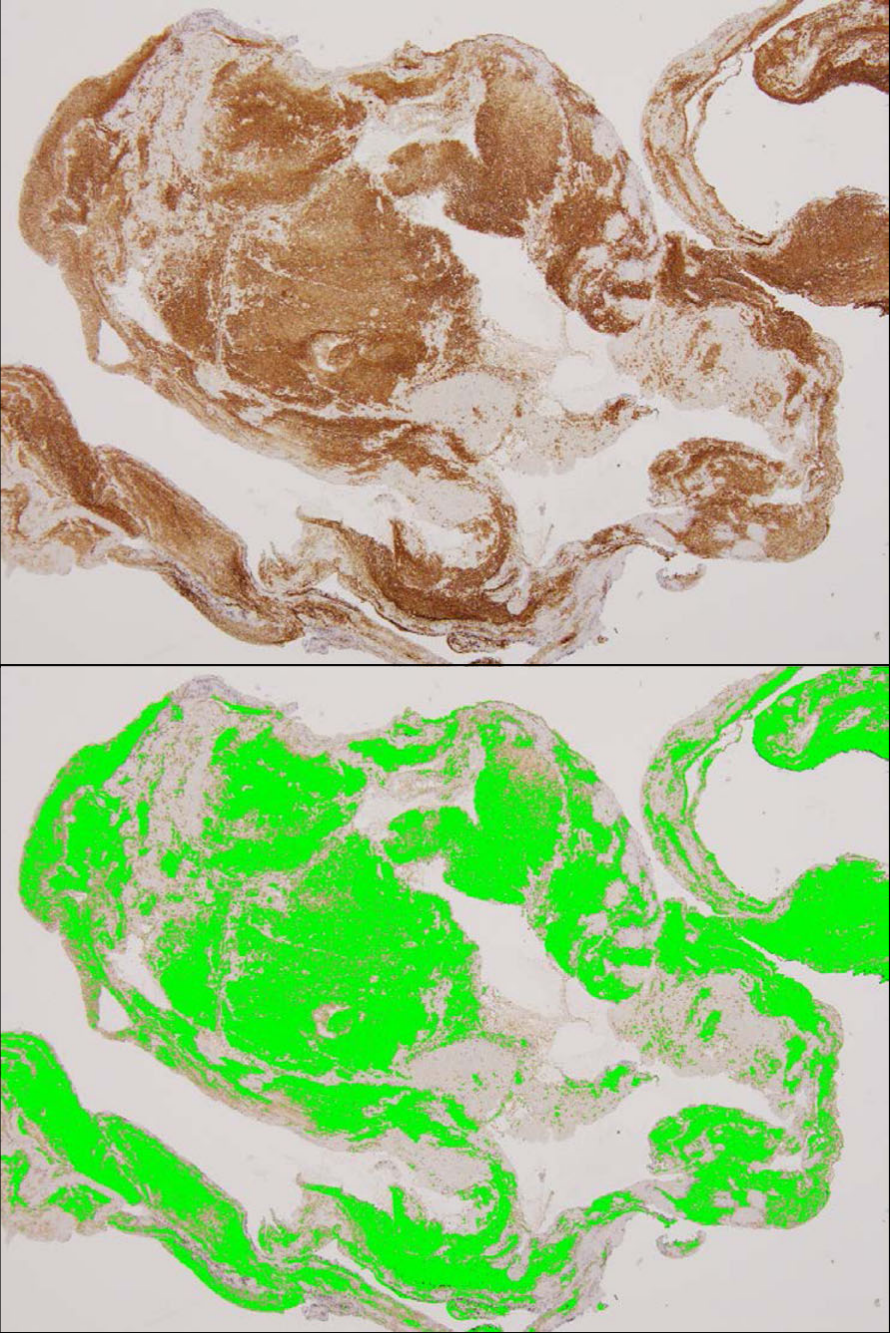
Representative immunohistochemical image of glycophorin A and its color-extracted image in image analysis. Immunopositive areas were extracted using specific protocols based on the color parameters of hue, lightness, and saturation. The data are expressed as ratios (%) of the extracted light *green* areas in the areas of thrombus

The sizes of the thrombi were measured in sections under a microscope using the NIS-Element-D 3.2 image analysis software (Nikon, Tokyo, Japan). Areas that were immunopositive for CD34, fibrin, integrin α2bβ3, glycophorin A, and SMA were semiquantified using the Win Roof color image analysis software (Mitani, Fukui, Japan) (Fig. 2) [10]. These areas are expressed as the ratios of positively stained areas per thrombus area. The number of granulocytes without lytic change was counted in the five most cellular fields under a 20× objective lens, and is expressed as the number per mm^2^. The immunopositive cell numbers for CD68, CD163, and CD206 in venous thrombi were counted in the five most heavily stained fields under a 20× objective lens. Cell density is expressed as the number of immunopositive cells per mm^2^.

### Statistical analysis

All data are presented as medians and interquartile ranges. Differences between individual groups were tested using the Kruskal–Wallis test with Dunn’s multiple comparison tests (GraphPad Prizm 6; GraphPad Software Inc., San Diego, CA, USA). The relationships between factors were evaluated using Spearman’s rank correlation coefficients. Values of *P <* 0.05 were considered significant.

## Results

### Macro- and microscopic findings from aspirated venous thrombi

**Fig. 3:**
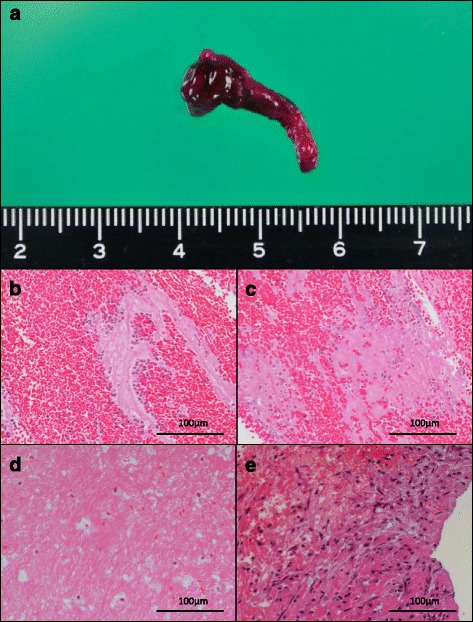
Representative macro- and microscopic images of aspirated deep vein thrombi. **a**. Representative macroscopic image of an aspirated thrombus. The aspirated thrombus is *red* or *mixed red* and *white*. **b**. Representative image of a fresh thrombus composed of erythrocyte-rich areas, eosinophilic granular or fibrinous areas, and polymorphonuclear or mononuclear leukocytes. Neutrophils are mainly accumulated at the border of the erythrocyte-rich and eosinophilic areas (9 days after onset). **c**. Representative image of lytic changes, including the loss of cellular morphology, karyolysis, and karyorrhexis (9 days after onset). **d**. Representative image of macrophage-like cells (60 days after onset). **e**. Representative image of an organizing reaction showing fibroblastic/myofibroblastic proliferation, leukocytic infiltration, and matrix deposition (33 days after onset). Hematoxylin and eosin stain (**b**–**d**)

The aspirated thrombi were red or mixed red and white (Fig. 3a). The thrombi were composed of erythrocyte-rich areas, eosinophilic granular or fibrinous areas, and granulocytes or mononuclear leukocytes (Fig. 3b). Neutrophils were mainly accumulated at the border of erythrocyte-rich areas and eosinophilic granular or fibrinous areas (Fig. 3b). The thrombi showed various degrees of cell lytic change (Fig. 3c) and infiltration of macrophage-like cells in part (Fig. 3d). One half of the thrombi focally exhibited organizing reactions with infiltration of mononuclear leukocytes (Fig. 3e).

### Immunohistochemical findings from aspirated thrombi

**Fig. 4:**
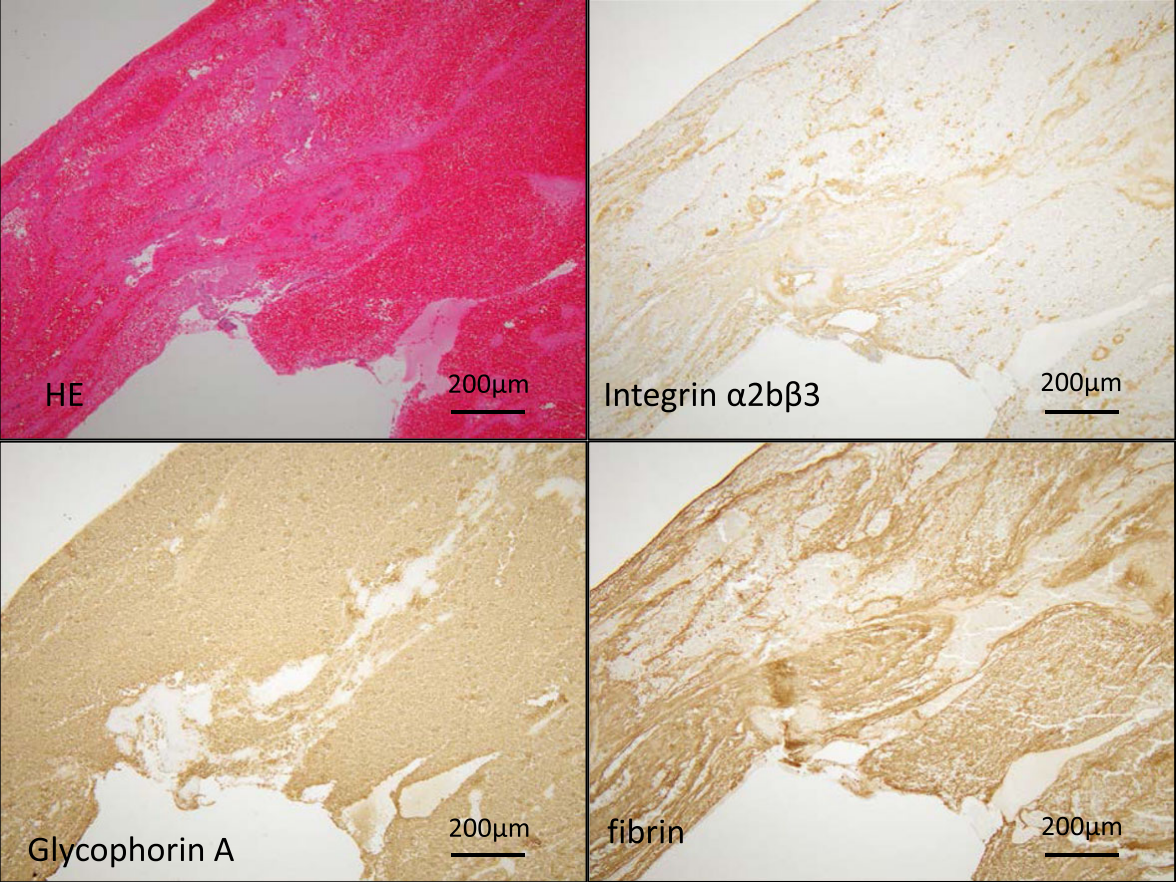
Representative immunohistochemical images of an aspirated deep vein thrombus. The thrombus is stained with hematoxylin and eosin (HE) and antibodies for glycophorin A, integrin α2bβ3, and fibrin. The thrombus is rich in glycophorin A and fibrin

All venous thrombi were immunopositive for glycophorin A, platelet integrin α2bβ3, and fibrin (Fig. 4, Table 1). Among them, glycophorin A- (*p <* 0.0001) and fibrin-immunopositive areas (*p <* 0.01) were larger than those of the integrin α2bβ3-immunopositive area (Table 1). The thrombi had small foci of CD34- and SMA-immunopositive areas (Table 1). Flat CD34-positive cells covered the surface of thrombi and formed microvessels. CD34 and SMA-immunopositive areas were positively correlated (*r =* 0.73, *p <* 0.01). There were CD68- and CD163-immunopositive mononuclear cells and stellate cells in all thrombi. The numbers of CD68- (*p <* 0.01) and CD163-positive cells (*p <* 0.0001) were significantly greater than those of the CD206-positive cells (Table 1). Immunopositive areas and cell numbers were not different in patients with (*n =* 13) or without heparin and/or thrombolytic therapy (*n =* 3).

**Table 1:** Components of deep vein thrombi and their relationships with the time after onset

	Median (interquartile range)	Correlation coefficient	*p* value
fibrin (%)	31 (24–41)	−0.11	0.67
glycophorin A (%)	46 (34–49)	−0.76	<0.001
integrin α2bβ3 (%)	14 (8–18)	0.04	0.89
CD34 (%)	0.06 (0.02–0.29)	0.35	0.18
SMA (%)	0.01 (0.001–0.07)	0.23	0.38
Granulocytes (/mm^2^)	204 (57–501)	–0.18	0.49
CD68 (/mm^2^)	263 (122–460)	0.60	<0.05
CD163 (/mm^2^)	308 (221–553)	0.64	<0.01
CD206 (/mm^2^)	67 (30–92)	0.44	0.09

*SMA* smooth muscle actin

Data were analyzed using Spearman’s rank correlation coefficient

### Relationships between the time after onset and cellular and molecular parameters

**Fig. 5:**
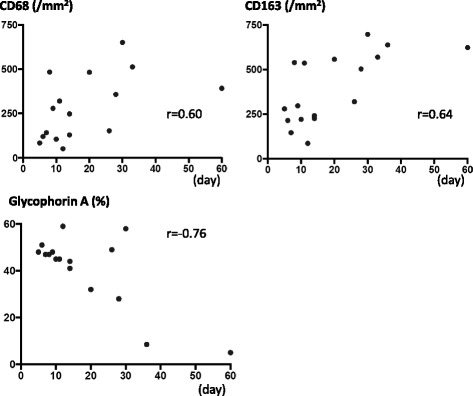
Relationships of the time after onset with CD68- and CD163-immunopositive cell numbers and glycophorin A-immunopositive areas

Table 1 shows the relationships between the time after onset and cellular and molecular parameters. CD68- and CD163-immunopositive cell numbers were positively correlated with the time after onset, while the glycophorin A-immunopositive area was negatively correlated with the time after onset (Table 1, Fig. 5). There were no significant correlations of the time after onset with granulocyte number, CD34-immunopositive area, and SMA-immunopositive area.

### Localization of CD163-positive cells in DVT

**Fig. 6:**
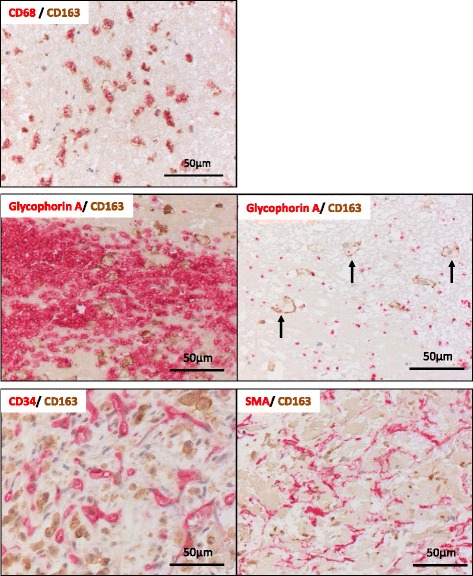
Localization of CD163 in aspirated deep vein thrombi. Double immunohistochemical staining for CD68 and CD163 (upper panel), glycophorin A and CD163 (middle panels), and CD163 and CD34 or SMA (lower panels). Expression of CD163 is visualized as a *brown* stain. Expression of CD68, glycophorin A, CD34, and SMA are visualized as a *red* stain. Most of the CD163-immunopositive cells (*brown*) are immunopositive for CD68 (*red*) (upper panel). CD163-immunopositive cells in erythrocyte-rich areas and phagocytosis of erythrocyte fragments can be seen (arrows, middle panels). Oval or stellate CD163-immunopositive cells in CD34- and SMA-immunopositive organizing areas can be seen (lower panels)

Double immunohistochemical staining showed colocalization of CD68- and CD163-positive cells, accumulation of CD163-positive cells in erythrocyte-rich areas, and phagocytosis of erythrocyte fragments. Oval or stellate CD163-positive cells were also accumulated in CD34- and SMA-immunopositive areas (Fig. 6).

## Discussion

The present study showed that the majority of macrophages expressed CD163 in aspirated thrombi from patients with DVT and that CD163 macrophages were closely distributed in erythrocyte-, CD34-, and SMA-immunopositive cell-rich areas. Additionally, CD163 macrophage or erythrocyte contents were positively or negatively correlated with the time after onset.

The traditional view is that deep vein thrombi are composed of erythrocytes with a large amount of fibrin and relatively few platelets [11]. This view is based on hematoxylin and eosin staining. Takahashi et al. [12] immunohistochemically examined the attached portions of deep vein thrombi in autopsy cases of venous thromboembolism and found that fibrin and erythrocyte contents tended to exceed the platelet content. However, there were no significant differences among the contents in the thrombi. Fibrin, erythrocyte, and platelet contents were consistently present in aspirated thrombi, and the thrombi were rich in erythrocytes and fibrin. These findings support the traditional view. Our findings also suggested that thrombus content differed in the portion of DVT, because aspirated thrombi were mainly obtained from the proximal portion of the thrombi. Additionally, we identified a time-dependent change in erythrocyte content, but not fibrin and platelet contents, within 60 days after onset.

Monocytes/macrophages are cellular components of venous thrombi. These cells appear around the edges of ligation-induced thrombi and become evenly distributed through the thrombi as resolution progresses in the rat [13]. Macrophage content time-dependently increases in experimental venous thrombi in the rat within 21 days [13] and peak at 7 days in mice [14] and 14 days in rabbits [15]. The positive relationship between macrophage content and the time after onset in aspirated thrombi is compatible with that of experimental studies. This relationship suggests that the thrombus resolution process is delayed in humans.

CD163 is a scavenger receptor for the hemoglobin–haptoglobin (HbHp) complex in macrophages. Several microenvironmental factors affect CD163 expression. Interleukin (IL)-6, IL-10, and glucocorticoids upregulate the expression of CD163 in monocytes/macrophages, while tumor necrosis factor-α, IL-1β, interferon-γ, lipopolysaccharide, IL-4, IL-13, oxidative stress, and hypoxia downregulate this expression [16]. High concentrations of HbHp complex also induce CD163 expression with an increase in secretion of IL-6 and IL-10 [17]. Hemoglobin that is released from lytic erythrocytes in venous thrombi is likely to enhance HbHp complex formation and upregulates CD163 expression in thrombus-associated macrophages. Previous studies have suggested a role for the binding of the HbHp complex to CD163 in anti-inflammatory responses of macrophages [18]. Macrophages in M2 or M2-like activation mode are related to resolution of inflammation, tissue repair, and remodeling via anti-inflammatory cytokine and angiogenic factor production, and extracellular matrix digestion and deposition [7]. Macrophages modulate the function and growth of human mesenchymal stem cells [19]. Therefore, interactions with endothelial cells, fibroblasts, and tissue stem cells could be important components of the role of macrophages in angiogenesis and tissue repair. CD163 macrophages are frequently localized to areas of interstitial fibrosis, collagen deposition, and accumulation of SMA-positive myofibroblastic cells in chronic kidney allograft injury [20]. The interactions between endothelial cells, myofibroblasts/SMCs, and CD163 macrophages are not fully understood. However, erythrophagocytosis and the nearby distribution of CD163 macrophages, endothelial cells, and myofibroblasts/SMCs suggest that CD163 macrophages play a role in venous thrombus resolution/organization.

CD206 is known as a mannose receptor C type 1 that recognizes sugars on microorganisms and some endogenous glycoproteins. CD206 and CD163 are upregulated in human atherosclerotic lesions with intraplaque hemorrhages [21]. However, CD206 expression is not prominent in DVT. In contrast to CD163, CD206 is upregulated by IL-4 and IL-13 [7]. These results suggest that there are microenvironmental differences between thrombus organization and hemorrhagic, atherosclerotic plaques.

There are small foci of CD34- and SMA-immunopositive areas in nearly all thrombi. Experimental stasis-induced venous thrombi have shown that microvessels or myofibroblasts and/or SMCs appear at 5 and 7 days, respectively, and that these areas time-dependently increase [22]. Although we observed a positive relationship between CD34- and SMA-immunopositive areas, these positive areas did not significantly correlate with the time after onset. This discrepancy could be due to the dynamic process of human DVT and the small foci of CD34- and SMA-immunopositive areas (Table 1) of the aspirated thrombi in this study.

There are several biomarkers for diagnosis of DVT, active coagulation, and post-thrombotic syndrome. Because CD163 macrophage and erythrocyte contents were positively or negatively correlated with the time after onset in our study, soluble CD163 (sCD163) and some erythrocyte markers might predict acuity of DVT. CD163 is shed from the macrophage surface into the circulation as sCD163, and sCD163 levels increase during inflammation and macrophage activation [23]. Therefore, sCD163 could be a possible marker for evaluating thrombus organization in patients with DVT without inflammatory diseases. Suades et al. [24] reported higher erythrocyte (glycophorin A-positive)-derived microparticle levels in acute coronary syndrome compared with control subjects. Because the thrombus size in DVT is larger than coronary thrombus size, glycophorin A-positive microparticles could be a possible marker for the acute phase of DVT.

This study has several limitations. We estimated the time of onset based on clinical symptoms and a medical interview. However, the onset of symptoms does not always indicate the onset of thrombus formation. This discrepancy might affect quantification of immunostaining results. Our findings from aspirated thrombi only represent a part of DVT. This could affect the value of the immunopositive area, especially with CD34 and SMA. In our study, histological samples did not provide information about the dynamic processes of thrombus formation and thrombolysis. Immunopositive areas and cell numbers were not different in patients with or without heparin and/or thrombolytic therapy, although we cannot deny β error due to the low power in each group. Although the sample number was small, the findings regarding aspirated thrombi from patients with DVT are important for better understanding the pathophysiology of DVT.

## Conclusions

We histologically and immunohistochemically analyzed aspirated thrombi that were obtained from patients with DVT. Our study shows CD163- and glycophorin A-immunopositive areas are positively or negatively correlated with the time after onset. CD163 and erythrocyte contents and related products could be markers for evaluating the age of DVT. CD163 macrophages might play a role in the organization of DVT.

## Abbreviations

DVT: Deep vein thrombosis
HbHp: Hemoglobin–haptoglobin
IL: Interleukin
SMA: Smooth muscle actin
